# Breast Mass Detection in Digital Mammogram Based on Gestalt Psychology

**DOI:** 10.1155/2018/4015613

**Published:** 2018-05-02

**Authors:** Hongyu Wang, Jun Feng, Qirong Bu, Feihong Liu, Min Zhang, Yu Ren, Yi Lv

**Affiliations:** ^1^Department of Information Science and Technology, Northwest University, Xi'an 710127, China; ^2^School of Mathematics, Northwest University, Xi'an 710127, China; ^3^Department of Breast Surgery, School of Medicine, The First Affiliated Hospital of Xi'an Jiaotong University, Xi'an 710061, China; ^4^National Local Joint Engineering Research Center for Precision Surgery and Regenerative Medicine, Xi'an Jiaotong University, Xi'an 710061, China

## Abstract

Inspired by gestalt psychology, we combine human cognitive characteristics with knowledge of radiologists in medical image analysis. In this paper, a novel framework is proposed to detect breast masses in digitized mammograms. It can be divided into three modules: sensation integration, semantic integration, and verification. After analyzing the progress of radiologist's mammography screening, a series of visual rules based on the morphological characteristics of breast masses are presented and quantified by mathematical methods. The framework can be seen as an effective trade-off between bottom-up sensation and top-down recognition methods. This is a new exploratory method for the automatic detection of lesions. The experiments are performed on Mammographic Image Analysis Society (MIAS) and Digital Database for Screening Mammography (DDSM) data sets. The sensitivity reached to 92% at 1.94 false positive per image (FPI) on MIAS and 93.84% at 2.21 FPI on DDSM. Our framework has achieved a better performance compared with other algorithms.

## 1. Introduction

Breast cancer is responsible for 23% of all cancer cases and 14% of cancer-related deaths amongst women worldwide [1]. Mammography is a reliable and trustworthy tool for early detection of breast cancer [2, 3]. Early detection of potential abnormalities could generate a recommendation for further examination [4]. Current progress has shown that computer-aided detection (CAD) systems can assist doctors in finding breast masses from digitized mammograms at an early stage, which greatly improves doctor's working efficiency [5]. Efficient CAD systems have potential to reduce the breast biopsies and release radiologists from heavy workload [6, 7].

However, CAD for breast mass detection is a challenging task due to the varying sizes, shapes, and appearances as well as densities of masses [8, 9]. Conventional methods for breast mass detection mainly rely on the threshold values [10] or mass templates [11] based on various kinds of filter operators. However, the false-positive results are still very high [12, 13]. The threshold methods based on gray-level images or various filtered images consider only the simple brightness of masses. Although there are ongoing research studies trying to model templates that use the general geometric properties of the masses [11], these are always complex, multiparameter models which are not applicable to all masses with various sizes and shapes.

**Figure 1:**
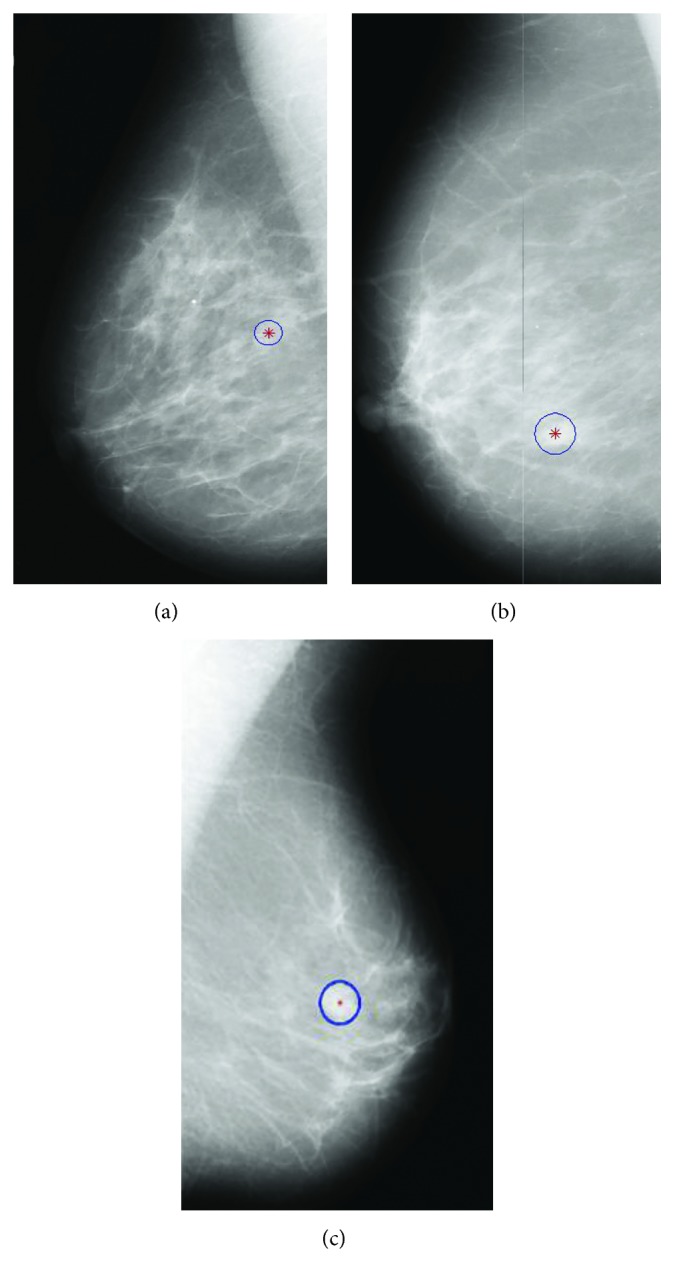
Sample images from MIAS data set [17].

In order to cope with the problems above, some researchers get inspirations from the doctors' detection process. They use the visual salience to locate the suspicious lesions [14, 15]. Visual saliency models human beings' ability that perceives salient features in an image. In computer vision, these visual saliency-based methods compute probabilistic maps of an image where the pixels are very different from surrounding regions [16]. These methods greatly simplify the process of mass detection. For example, Tourassi et al. [14] proposed a novel similarity measure by incorporating the Gaussian salient map of image pixels. Agrawal et al. [16] proposed an automatic mass detection algorithm using the graph-based vision saliency (GBVS) map. However, most of these methods are derived from natural scene statistics while the characteristics of medical images are different. In fact, they only consider the size and brightness of suspicious regions, which is not enough for mass detection, as shown in Figure 1. Some masses are so small or they adhere tightly to glandular tissue. The visual significance is relatively low, resulting in a high false positive.

At present, deep learning [18] has been shown to be consistently producing higher performance compared with traditional machine learning methods [19]. Directly distilling information from training samples and convolutional neural networks (CNNs) [20] have been successfully applied to some medical tasks: for example, breast mass detection/diagnosis [21, 22], segmentation of the left ventricle [23], and classification of skin cancer [19]. Dhungel et al. [24] presented an automated mass detection method using a cascade of deep learning and random forest classifiers. Kooi et al. [25] have shown that a CNN trained on a large data set of around 45,000 images outperforms a state-of-the-art system in CAD. Most of these methods work well on large data sets but exhibit certain limitations on small data sets because they need to decide a large number of parameters [26]. Therefore, the traditional machine learning method is still valuable in some aspects, like data mining based on small samples [27], integration of multiple knowledge [28], and so on.

Studies have shown that the recognition of doctors plays an important role in lesion detection of radiology [29, 30]. Gestalt psychology tries to understand the laws of our ability to acquire and maintain meaningful perceptions in an apparently chaotic world [31]. Meanwhile, the theory has been validated on extensive experiments, which are performed on neatly organized dot lattices on a screen. These dots share many similarities with pixels in medical images [32, 33]. Hence, we consider incorporating visual perception properties described by the Gestalt psychology framework into mass detection. Inspired by Gestalt psychology, the Gestalt framework covers sensation (bottom-up) and perception (top-down), which are also collectively called recognition [34]. The theory aims to formulate visual rules according to which perceptual input is organized into unitary forms. The Gestalt theory includes the following principles: proximity, similarity, continuity, symmetry, closure, simplicity, and so on [34]. These visual rules can be used to help doctors to distinguish cancer masses from normal tissues.

Inspired by the framework of Gestalt theory, we propose to apply visual rules to medical image analysis. More exactly, we present an automatic mass detection framework based on Gestalt psychology. It contains three modules: sensation integration, semantic integration, and validation. In each module, a series of mathematical and calculation models for visual rules are presented. The proposed automatic mass detection method integrates human cognition properties and the visual characteristics of breast masses. To the best of our knowledge, combining bottom-up sensation and top-down recognition of the radiologist has not been attempted before. Experimental results demonstrated that the proposed method has yielded better performance than other algorithms.

## 2. Mass Detection Framework Inspired by Gestalt Psychology

In this paper, we propose to incorporate the visual perception properties into breast mass detection. First, the characteristics of mammogram reading by radiologists are analyzed as per Gestalt psychology. Second, a framework for automatic detection of masses is proposed. All visual rules in the framework are quantified through mathematical methods.

### 2.1. Analysis of Mammogram Screening under the Gestalt Framework

In most cases, screening mammogram is a process of discovery, detection, and diagnosis by the radiologist [35]. The diversity of mammogram tissues brings many problems to mass detection. Radiologists are professional analysts who spend thousands of hours refining their abilities of detecting lesions in medical images. They gain a lot of experience in the learning process. According to Gestalt psychology research, radiologists read the images with the eyes and the brain. The visual rule plays an important role in recognizing masses from mammogram screening.

**Figure 2:**
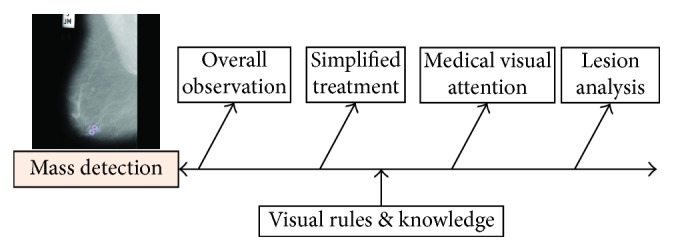
The clinical diagnosis of breast mass by the radiologist.

In clinical practice, radiologists tend to analyze medical images from overall impression down to individual parts for single-read mammography (or top-down), as shown in Figure 2. At the beginning, radiologists go through the mammogram and then focus on the highly suspicious areas. In vision psychology, the eyes can only accept a small number of associated units. If a Gestalt framework contains too many unrelated units, the eyes try to simplify it and combine the units into a big unit that can be processed easily. That is, our brain tends to combine and simplify the units [31]. Then, all the suspicious areas (called regions of interest, ROIs) will be further analyzed to get the masses. Many factors need to be considered by radiologists, such as morphology, density, and correlation with surrounding tissue. Generally speaking, a mass is a generic term indicating a localized swelling protuberance or lump in the breast [11].

### 2.2. The Framework of Automatic Mass Detection Based on Gestalt Framework

**Figure 3:**
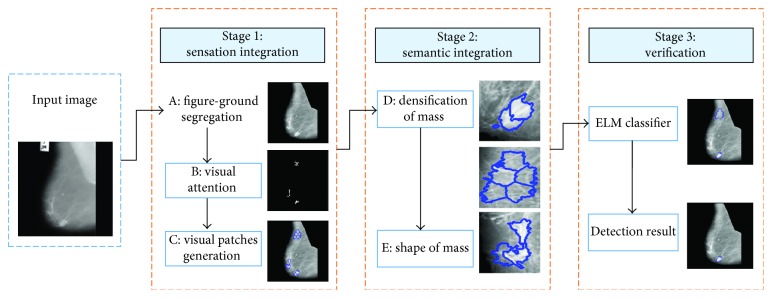
The framework of the proposed approach. It can be divided into three stages including sensation integration, semantic integration, and verification. Visual rules used in the framework are modeled and indicated with the labels A, B, C, D, and E.

Inspired by the clinical practice, a mass detection method based on Gestalt framework is proposed in this paper (Figure 3). We divide the framework into three stages including sensation integration, semantic integration, and verification. It can be viewed as a combination of bottom-up sensation and top-down recognition methods. For each part, there are various visual rules based on Gestalt psychology and morphological characteristics.

#### 2.2.1. Stage 1: Sensation Integration

In the initial stage, the visual sense of the radiologist plays an important role in mass detection. The attention is a process of selecting and getting visual information from pixels of the image (bottom-up) [36].

Observation 1: *From doctors' experience, the mass areas are located in the breast zone and are always more salient than the surrounding area. Inspired by the Gestalt framework, three rules are defined for image simplification, including figure-ground segregation, visual patches generation, and visual attention.*

*(1) Figure-Ground Segregation.* The principle of figure-ground segregation is one of the basic cognitive principles [37]. When applied to a mammogram, this principle supposes that the background does not contain any valuable information and is neglected by radiologists, obviously a simplified treatment. As a result, some existing methods propose to separate the figure from the background [38]. The mammograms have low contrast and still have noise in the background such as tape markings and labels (as shown in Figure 3). We use an adaptive global threshold to compute the outline of the breast region [39]. Based on the morphological analysis, an image enhancement method is adopted [40], which can effectively suppress the background and enhance the features of masses on mammograms simultaneously. Meanwhile, the pectoral muscle is removed [41].

*(2) Visual Attention in the Medical Image.* As we all know, the mass areas are always “brighter” than the surrounding areas. That is, the highlighted region attracts more visual attention than the background region when doctors browse the mammogram. It is called prominence in Gestalt psychology. A simple method is to predefine a threshold value for a breast image. However, this approach is unfavorable as there is a large variance between tissues in mammogram. Following [42], opening operation is adopted to find the focal area in the mammogram:

(1) $$\begin{array}{c} I_{\Phi }=I\circ \Phi =\left(I⊗\Phi \right)⊕\Phi , \end{array}$$

where ∘ is the morphological opening operation, ⊗ is the erode operation, and ⊕ is the dilation operation. The morphological opening operation consists of two steps in our method. Firstly, the original image *I* is eroded (⊗) with the structural element Φ. Φ is created by a flat disk-shaped structuring element with the specified radius of 6 pixels in the experiments. Secondly, dilation (⊕) is performed on the eroded image to produce the final reconstructed image (*I*_Φ_). And then, we perform regional maximum on the reconstructed image, which retrieves all the salient regions without overselecting any of them.

**Figure 4:**
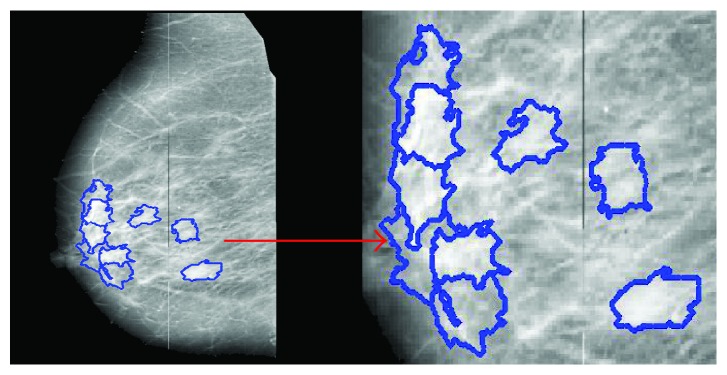
Visual patches based on Gestalt psychology.

*(3) Visual Patches Generation.* Gestalt theory aims to formulate some rules according to which the perceptual input is organized to unitary forms such as wholes, groups, or gestalt. The most common method is to group the similar or proximate object together. Inspired by the concept of “superpixel” [43], the basic processing units (visual patches) are generated by using our previous work [44]. In [44], the abdominal computed tomography (CT) image is divided into many visual patches as per the law of similarity evaluated by both intensity and spatial distance. Now, the proposed method is applied on mammograms (Figure 4). Visual patches are generated by clustering pixels based on both intensity similarity and spatial proximity. Firstly, *K* cluster centers are set to divide the image into several rectangular patches. Then, we use the following similarity index *D*_s_ (2) to cluster pixels in mammograms:

(2) $$\begin{array}{c} D_{s}=\frac{\mu }{S}d_{xy}+d_{g}, \end{array}$$

where *d*_*xy*_ is the spatial proximity, *I*_Φ_=*I*⁡∘⁡Φ=(*I*⁡⊗⁡Φ)⁡⊕⁡Φ, which is calculated by Euclidean distance on image plane, *d*_*g*_ is the intensity distance, and *μ* is the parameter of the pixel compactness [44]. *S* is a constant which balances the spatial proximity in image gray space, which is set as 25 in the experiment. The generated visual patches act as the basic processing units in the mass detection.

Visual patches are generated only in the salient positions which reduce the computation expense greatly as shown in Figure 4. These visual patches are the basic processing and analysis units in our algorithm. Suppose we have *M* visual patches which meet the condition of visual attention. *U*={*P*_1_, *P*_2_,…, *P*_*M*_} is the set of *M* visual patches in a mammogram, and we assume that all of these patches are candidate masses in the sensation phase. Meanwhile, we define another set *N*=*ϕ* to store the patches which is regarded as normal tissue.

#### 2.2.2. Stage 2: Semantic Integration

After the first stage (sensation integration), there are still many false-positive patches in *U* after the first stage (sensation integration). According to morphological characteristics of breast tissue, we introduce the semantic integration to further distinguish the masses patches from normal tissue. Kinds of semantic features of visual patches are used to help separate the mass from the normal tissue. The semantic integration can be regarded as a top-down recognition process [45].

Observation 2: *Normal tissue is always rich in glands and has poorer or lower density than that of the mass region. In addition, the shape of masses tends to be round and oval* [46, 47]. *Masses and glandular tissues have very different shapes. Following the Gestalt principle, we propose two rules based on morphological characteristics of masses, including densification and shape.*

**Figure 5:**
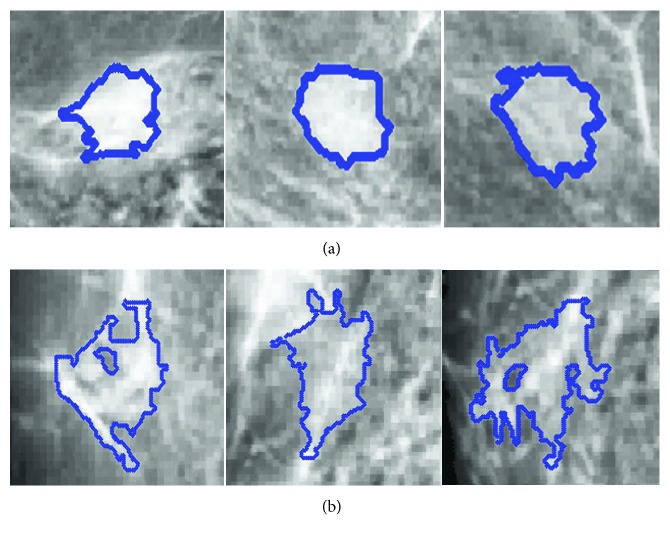
The densification of different patches. Visual patches of (a) mass and normal tissue.

*(4) Densification of Mass.* Eltonsy et al. [11] showed that the growth of a mass disrupted the normal breast parenchyma structure and formed a focal activity area called “focal seeds” in our research. According to the Gestalt principle, the law of similarity indicates that elements are perceptually grouped together if they are similar. The focal seed is regarded as an entirety because it has a strong self-similarity as shown in Figure 5, whereas normal tissue sometimes has a poor densification and even forms some holes in the visual patch (Figure 5). The main reason is that normal tissue contains rich glands that affect the consistency of a given visual patch. The visual patches of masses exhibit homogeneity, whose solidity values are very high [48]. Here, the densification is defined as

(3) $$\begin{array}{c} \begin{array}{c} \text{Dens}=\sum_{i}\sum_{j}\frac{\left(ij\right)M\left(i,\;j\right)-u_{i}u_{j}}{\sigma_{i}\sigma_{j}},\\ u_{i}=\sum_{i}\sum_{j}i\cdot M\left(i,j\right),\\ u_{j}=\sum_{i}\sum_{j}j\cdot M\left(i,j\right),\\ \sigma_{i}^{2}=\sum_{i}\sum_{j}M\left(i,j\right){\left(i-u_{i}\right)}^{2},\\ \sigma_{j}^{2}=\underset{i}{\sum }\underset{j}{\sum }M\left(i,j\right){\left(i-u_{j}\right)}^{2}, \end{array} \end{array}$$

where *M*(*i*, *j*) is the gray-level co-occurrence matrix of the visual patch *P*(*i*, *j*) and *u* and *σ* are the mean value and variance for each visual patch *P*, respectively. The bigger the Dens (densification) is, the greater the probability of it being a tumor; otherwise, it is more likely to be the normal tissue. Therefore, a threshold *T*_dens_ is assigned based on the results of statistical analysis. Each visual patch in the candidate set *U* is detected, and the false-positive rate of visual patches is reduced as shown in (4), where *N* is the set to store visual patches of normal tissue:

(4) $$\begin{array}{c} \begin{array}{l} P_{i}\in N\text{ if Dens}\left(P_{i}\right)<T_{\text{dens}},\\ P_{i}\in U\text{ if Dens}\left(P_{i}\right)\geq T_{\text{dens}}, \end{array} \end{array}$$

**Figure 6:**
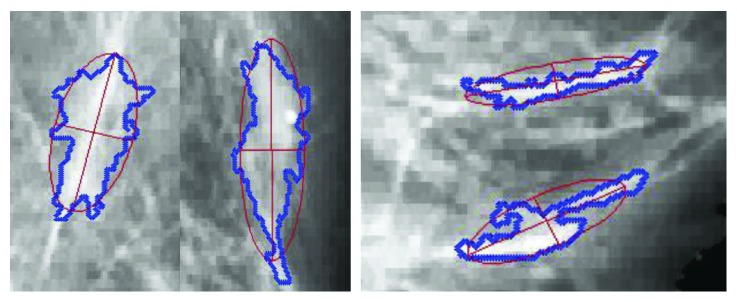
Visual patches with the glandular.

*(5) Shape of Mass.* As we all know, an abundance of glands exist in the breast. They are radically arranged from the center of nipple, like a crown, occupying a large part of the mammogram. The brightness of the gland is most similar to the tumor tissue, and this causes high false-positive results in various CAD systems [16]. But the shape of visual patches which contain glandular tissue is always a long strip as shown in Figure 6.

Comparatively, the visual patches of tumor tend to be round and oval [46] (Figure 5). In addition, the focused visual patch is positioned only in the center area of mass rather than its edge. The main reason is that many masses have no obvious margins surrounded by glandular tissues. The continuity law of Gestalt states that elements of objects tend to be grouped together and integrated into perceptual wholes. In other words, the patches containing glandular tissue can be easily perceived with the distribution of continuity. In that case, the linear patches can be filtered from the candidate visual patches.

Here, we bring in the concept of eccentricity to restrict the shape of each visual patch, which is an important index in ellipse. An ellipse fitting algorithm is used for each visual patch edge to get the eccentricity (Figure 6). The elliptic equation is defined as

(5) $$\begin{array}{c} A^{\prime }x^{2}+B^{\prime }xy+C^{\prime }y^{2}+D^{\prime }x+E^{\prime }y+F^{\prime }=0. \end{array}$$

There are 6 parameters in (5), that is, *A*′, *B*′, *C*′, *D*′, *E*′,  and  *F*′. They can be estimated by using the least-squares method according to the edge points of each patch. Then, the eccentricity is defined using the long and minor axis of the fitted ellipse:

(6) $$\begin{array}{c} \begin{array}{l} E\left(P_{i}\right)=\frac{\sqrt{a_{i}^{2}-b_{i}^{2}}}{a_{i}}=\sqrt{1-{\left(\frac{b_{i}}{a_{i}}\right)}^{2}},\\ a_{i}=2\sqrt{\frac{-2F^{\prime }}{A^{\prime }+C^{\prime }-\sqrt{{B^{\prime }}^{2}+{\left(A^{\prime }-C^{\prime }/F^{\prime }\right)}^{2}}}},\\ b_{i}=2\sqrt{\frac{-2F^{\prime }}{A^{\prime }+C^{\prime }+\sqrt{B^{\prime 2}+{\left(A^{\prime }-C^{\prime }/F^{\prime }\right)}^{2}}}}, \end{array} \end{array}$$

where *a*_*i*_ and *b*_*i*_ are margin axis and minor axis of the ellipse, respectively. *E*(*P*_*i*_) measures the circularity of the visual patch *P*_*i*_ and the range is from 0 to 1. Since we assume that the eccentricity value of a visual patch is significantly lower in tumors than that in normal tissues. A threshold *T*_e_ is defined to reduce the false-positive rate:

(7) $$\begin{array}{c} \begin{array}{l} P_{i}\in N\text{ if }E\left(P_{i}\right)\geq T_{e},\\ P_{i}\in U\text{ if }E\left(P_{i}\right)<T_{e}. \end{array} \end{array}$$

Based on the above rules, the number of candidate patches in set *U* is greatly reduced, while the sensitivity and specificity are very high which will be explained in detail in the experiment. Another advantage of our method is that the focused visual patches only lie in the center of tumors. It can lay the foundation for the further proceeding and analysis, such as mass segmentation and mass diagnosis.

#### 2.2.3. Stage 3: Verification: False-Positive Reduction Based on Texture Feature Classification

Observation 3: *The Gestalt theory shows that the information of visual perception is affected by observer's prior experience. Radiologists accumulate a great deal of knowledge to identify the texture of breast tissue. We propose a validation method based on texture of mass and extreme learning machine (ELM) classification method.*

The importance of texture features is obvious for mass detection. In this research, we have extracted intensity and gray-level co-occurrence matrix (GLCM) features on the candidate visual patches, including *N* dimensions for the patch *P*_*i*_, *F*(*P*_*i*_)={*g*_1_, *g*_2_,…, *g*_*N*_}. Among these features, two of them are the first-order texture feature, which is the mean of gray value and gray variance describing visual patches. Ten features are calculated from the gray-level co-occurrence matrix, namely, contrast, correlation, energy, homogeneity, standard deviation, inverse difference movement, kurtosis, skewness, entropy, and root mean square.

The candidate patches left in *U* serve as the seeds of region growing algorithm. ROIs are obtained by clustering these visual patches based on texture features *F*. The features of each ROI are represented by the average value of associated patches as shown in (8), where *M*_*j*_ is the total number of visual patches in ROI_*j*_:

(8) $$\begin{array}{c} F\left({\text{ROI}}_{j}\right)=\frac{\sum_{P_{i}\subseteq {\text{ROI}}_{j}}F\left(P_{i}\right)}{M_{j}}. \end{array}$$

Recently, extreme learning machine (ELM) has been extensively studied, and important progress has been made in both theories and practical applications. The ELM theory in [49] has proved that random feature mapping can provide universal approximation capability. The ELM has built some tangible links between machine learning techniques and biological learning mechanisms. It is an emerging learning algorithm for the generalized hidden layer feedforward neural network [49, 50]. Here, the ELM is used to simulate the final decision of doctors. Furthermore, it classifies the ROIs into normal and abnormal cases based on the texture feature extraction.

## 3. Experimental Results

### 3.1. Data Set and Parameter Setting

Our proposed method is tested on two publicly available data sets: MIAS [17] and DDSM [51]. A set of 257 mammograms of MIAS is used in the research. Among these images, 207 images do not contain any lesions while other 50 images have masses. The spatial resolution of image in MIAS is 50 *µ*m × 50 *µ*m, and grayscale intensity is quantized to 8 bits. The DDSM data set contains 210 images, in which 130 images contain masses and the other ones are normal mammograms. The images of DDSM have been resized to 1024 × 1024 pixels, and grayscale intensity is quantized to 8 bits in accordance with images in MIAS. In both MIAS and DDSM data sets, the mammograms containing masses have been annotated by expert radiologists, which are used for reporting the detection performance in our experiments. The ELM classification method divides visual patches into mass and nonmass candidates using 10-fold cross validation.

In our research, a series of indexes are used to quantitatively evaluate the effectiveness of our method. The performance indexes include sensitivity (Sens), false positives per image (FPI), homogeneity (Dens), and free-response receiver operating characteristic (FROC). These indexes are described below.

- Sens and FPI are computed as a region-based evaluation. If the ratio of the overlapping region of the ground truth and the computer-segmented mass region is larger than 50%, the region is considered as “True Positive Marks” or “Positive ROIs” [52]. Otherwise, it is considered as “False Positive Marks,” which is also called “Negative ROIs” in our experiments. For computer-aided systems, we would like the value of sensitivity to be as high as possible. Meanwhile, FPI should be low while guaranteeing high sensitivity [53].

(9) $$\begin{array}{c} \begin{array}{c} \text{Sens}=\frac{\text{number of true positive marks}}{\text{number of regions}}\\ \text{FPI}=\frac{\text{number of false positive marks}}{\text{number of images}}\text{.} \end{array} \end{array}$$

(b) In Section 3.2, we use homogeneity (Dens) to characterize the distribution of visual patches, which is defined as (3). It is based on the fact that every visual patch is an independent processing unit that should be homogeneous as per Gestalt rules [44]. The value of Dens ranges from 0 to 1. When patches are uniform, the value of Dens tends to be 1, while for nonuniform patches, the value tends to be 0.
(c) In Section 3.5, free-response receiver operating characteristic (FROC) [52] curve is used. The FROC curve is defined as the plot of sensitivity (Sens) versus the average number of false positives per image (FPI).

All numerical methods are performed using MATLAB 2012b software running on a desktop PC with a 2.50 GHZ CPU and 2G RAM. Different from data-driven algorithms like deep learning, our method does not need a large amount of data. The major reason is that it is designed based on human visual characteristic and radiologists' experience. There are all together three types of parameters in our method, which are (descending order of importance) the thresholds for visual rules, the parameters for generating visual patch, and some other parameters of the ELM classifier.

The medical images from different hospitals or different apparatuses may be completely different. Thus, the parameters of the method should be modified on different apparatuses or data sets. The thresholds for visual rules (medical image attention, densification of mass, and shape of mass) are crucial in the proposed method, which determine the number of suspicious regions, because three key parameters in the method are independent. By comparing the Sens and FPI in different thresholds, three parameters can be determined for new validation data. In addition, the parameters for visual patches generation and ELM classifier have less impact on detection performance than that for visual rules. If the input size of image is [*M*, *N*], then the initial size of the visual patch can be calculated by *M*⁡∗⁡*N*/*K*, where *K* is the cluster parameter for visual patches generation. That is to say, the bigger the value of *K* is, the smaller the size of visual patches is, and vice versa. The initial size of the visual patch is preferably less than the size of the entire breast mass. In this manuscript, both *M* and *N* is 1024, and *K* is set as 2000. ELM has been extensively studied, and it shows a good convergence speed and stability [49]. In our research, the parameters of ELM do not require careful adjustment. All parameters used in the experiments are shown in Table 1.

### 3.2. The Effectiveness of Visual Patches

**Figure 7:**
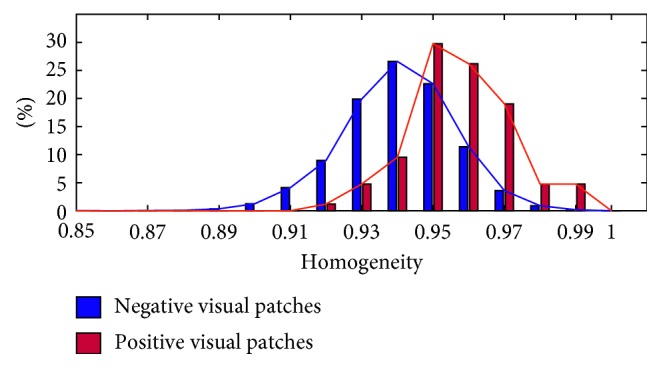
The statistical histogram of homogeneity of negative and positive visual patches.

The mass region patches on mammogram are called “positive patches,” and the normal tissue ones are “negative patches” in the following content.  Figure 7 shows the statistical histogram of homogeneity of visual patches both for negative and positive patches. It is obvious that the homogeneity of all patches is above 0.85, with positive patches having a homogeneity value above 0.9, center around the value 0.95. We can draw a conclusion that the use of visual patches ensures the semantic consistency of objects in ROIs, which lays a solid foundation for further research. Besides, the homogeneity of positive patches is a bit higher than that of negative patches, which supports the description of similarity rules defined in Section 2.2.2. As shown in Figure 7, the homogeneity distribution curves of positive and negative patches are similar to normal distribution. The two curves are distinguishable owing to the higher homogeneity of positive patches than that of the negative ones.

### 3.3. Mass Detection Performance

**Figure 8:**
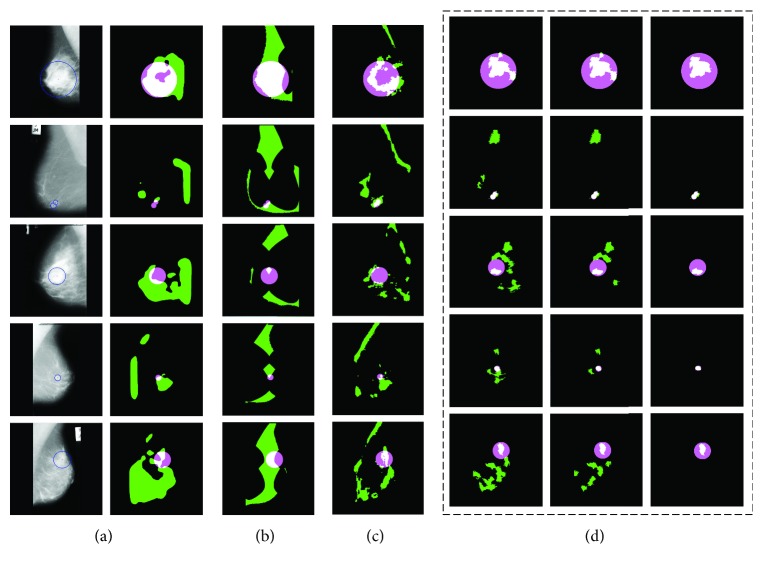
Sample results of the saliency algorithms. Green denotes the saliency region detected by these algorithms, pink represents the ground truth region containing mass, and white denotes the crossing region between green and pink. (a) Agrawal et al. [16], (b) Achanta and Süsstrunk [55], (c) Murray et al. [56], and (d) the three stages of our method. Stage 1: the fifth column is the detection result of sensation integration. Stage 2: the sixth column is the detection result of semantic integration. Stage 3: the last column is the final detection result (verification) of our method.

In the proposed framework, a series of visual rules have been defined, and finally, all the salient patches are saved (in set *U*). We compare the performance of our framework to three existing visual saliency algorithms: Agrawal et al. [16] (graph-based vision saliency), Achanta and Süsstrunk [54] (maximum symmetric surround saliency), and Murray et al. [55] (saliency estimation using a nonparametric low-level vision model), as shown in Figure 8.

In Figure 8, each row corresponds to the output of four algorithms, and the corresponding mammograms are shown in the first column. The detection results of three stages in the proposed method have been listed in Figure 8. It is obvious that our method outperforms other saliency algorithms for mammogram mass detection. At the stage of verification, the false-positive rate is further reduced and there is a bit impact on the mass region compared with normal tissue. We experimentally observe that Agrawal et al. [16] yielded a relatively good result that is in accordance with what is reported in [16]. However, it computes the saliency of a region with respect to its local neighborhood using the directional contrast. But it is invalid when the mass is surrounded by dense gland tissues as shown in the last row of Figure 8. However, the positive aspect of our method is that it combines visual cognitive theory with various morphological characteristics of masses. The advantages can be summarized as follows: (1) The detection method based on gestalt rules is able to detect masses of varying sizes, resulting in a low false-positive rate (the green region in Figure 8). (2) The salient visual patches of our method mostly lie in center of the ground truth regions. The detected results based on the proposed method can be used for further analysis, such as mass segmentation and diagnosis.

**Figure 9:**
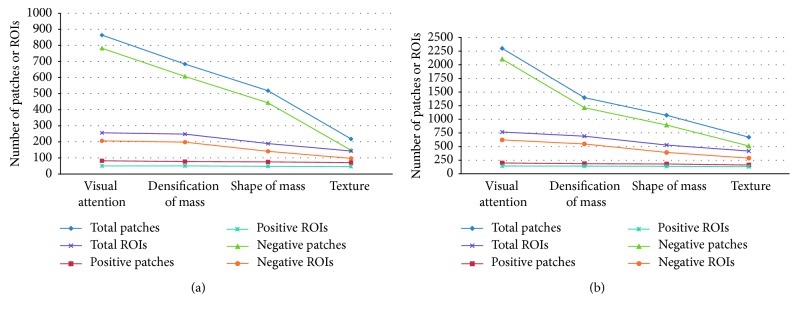
The number and percentage of patches/ROIs are counted for each step of our method: (a) plotted on the MIAS data set and (b) plotted on the DDSM data set.

Table 2 shows the overall performance of our proposed method on MIAS and DDSM data set. As shown in Table 2, the performances of three stages of our method are given. Masses are detected effectively, and Sens reaches 92% at 1.94 FPI and 93.84% at 2.21 on MIAS and DDSM data sets, respectively. As shown in Table 3, we can get extended statistic information on both the total number and percentage of patches and ROIs. Meanwhile, the performance curves are plotted in Figure 9. From Table 3 and Figure 9, the number of positive patches and positive ROIs remains largely unchanged, whereas the number of negative examples is greatly reduced as the detection stages are introduced. The performance curves show the similar change of detection performance for MIAS and DDSM. We can draw a conclusion that the positive visual patches can be preserved, and false-positive results are declined dramatically under the gestalt rules constraint.

### 3.4. Influence of the Breast Density for Mass Detection Performance

In general, masses in low-density breast are easily detected than masses in high-density breast [56]. To further evaluate the ability of our method, mass images with different densities are separated to test on the MIAS and DDSM data sets. The results are summarized in Table 4. There are three types of densities for MIAS, that is, fatty (*F*), fatty-glandular (*G*), and dense-glandular (*D*). Different from MIAS, the images in DDSM data set are divided into four categories based on breast density, that is, 1, 2, 3, and 4.

The quantity proportions of each subset with different densities are listed in the first row of Table 4. This table shows Sens and FPI of mass images with different densities. It shows that the algorithm has good performance and works well on different mass images. Looking at the MIAS results, the proposed method has superior performance on fatty (*F*) and fatty-glandular (*G*) breast images compared to the dense-glandular (*D*) images. Similarly, the method performs better in low-density (level 1 and level 2) images than high-density (level 3 and level 4) images on DDSM. Masses in low-density images usually have distinctive visual features compared with the tissue around it. Conversely, some visual patches with high density may cause erroneous judgment at the final recognition stage. So, the false-positive rates would increase when the data set includes many breast images with high density. In this section, the sensitivities for all lesions are 92% at 1.94 FPI on MIAS and 93.84% at 2.21 FPI on DDSM.

**Figure 10:**
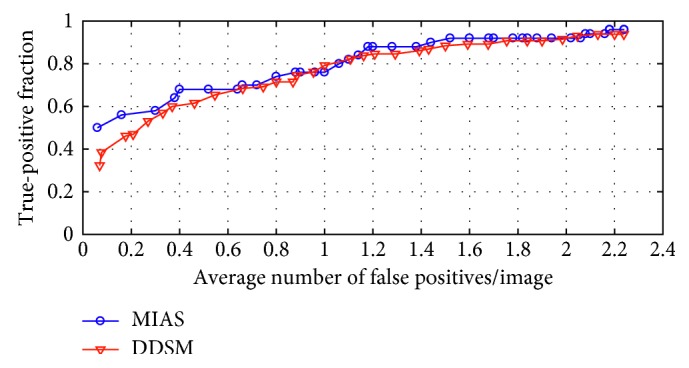
FROC curves of the proposed method on MIAS and DDSM data sets.

A common method for evaluating true-positive detection is free-response receiver operating characteristic (FROC) analysis [57]. It is a plot of operating points showing a tradeoff between the sensitivity rate and the average number of false positives per image. The complete FROC curves of our method are presented in Figure 10. The blue and red curves denote the detection results on MIAS and DDSM, respectively. We can get a favorable detection result when a false positive reaches 2 per image on the two data sets.

### 3.5. Comparison of Experimental Results

The mass saliency is introduced in the proposed framework. In order to evaluate the ability of our method, experiments were conducted with 10-fold cross validation on MIAS and DDSM. The performance is compared with other popular algorithms in terms of Sens and FPI. It is clearly shown in Table 5 that the proposed method has equivalent or even better accuracy than other algorithms. High sensibility and low FPI represent the good performance of an algorithm. We can get a series of Sens at different FPIs as shown in Figure 10. The sensibility reaches 92% at 1.94 FPI or 94% at 2.16 FPI on MIAS. Accordingly, the sensibility reaches 93.84% at 2.21 FPI or 94.6% at 2.66 FPI on DDSM. The comparative studying methods [52, 53, 58], following the similar pipeline, include two parts: image preprocessing and suspicious mass regions identification with different adaptive thresholds. In contrast, a sliding window scheme is utilized in [59], and texture features are modeled by kernel-based supervised hashing to get the mass location. Different from the sliding window in [59], visual attention of radiologists is used in our method. In the end, our method not only utilizes the advantages of machine learning approaches, but the visual saliency of mass is also modeled which achieves significant improvement in reducing false positives and sensitivity.

## 4. Conclusion

In this paper, we have proposed an automatic mass detection framework for digitized mammograms. The main contributions of our research can be summarized as follows: (1) The visual characteristic of radiologists is modeled based on the Gestalt theory. (2) An automatic mass detection framework is proposed which is in accordance with the doctors' visual perception. Some constraints are defined such as density and shape of visual patches. These parameters are probably used by experienced radiologists in detection/diagnosis masses and so on. (3) Our framework achieves a good performance both on MIAS and DDSM data sets.

Different from existing detection techniques, our methods use the visual patches as the basic processing unit. We focus on providing a more efficient and more innovative data analysis method for lesions detection than traditional methods. In our future work, more mammograms from different apparatuses will be tested to evaluate the performance of our proposal. We will further improve our method as per the feedback reports from more radiologists. Moreover, we would like to focus on expanding this research to the deep learning method.

## Figures and Tables

**Table 1:** Three types of parameters in the experiments.

Type	Parameters	Setting value
Visual rules	Medical visual attention threshold range: [0 1]	0.5
	Densification of the mass threshold range: [0 1]	0.93
	Shape of mass threshold range: [0 1]	0.86

Generation of visual patch	Image size	[1024, 1024]
	Cluster parameter, *K*	2000

ELM	Tradeoff parameters	*C*=10
	Kernel type	RBF
	Kernel parameter	0.01

**Table 2:** Mass detection performance (Sens and FPI) of different stages in our method.

Data	Index	Stage 1	Stage 2		Stage 3
		Visual attention	Densification	Shape	Texture
MIAS	Sens	100%	98%	96%	92%
	FPI	4.12	4.21	2.82	1.94

DDSM	Sens	99.23%	96.92%	96.15%	93.84%
	FPI	4.78	4.19	3.01	2.21

**Table 3:** Mass detection performance (number and percentage of patches/ROIs) of different stages in the proposed method.

Data	Unit	Stage 1		Stage 2				Stage 3	
		Visual attention		Densification		Shape		Texture	
MIAS	Total patches	864	100%	684	79.10%	518	59.95%	218	25.23%
	Positive patches	82	100%	77	93.9%	75	91.46%	71	86.59%
	Negative patches	782	100%	607	77.62%	443	56.67%	147	18.80%
	Total ROIs	256	100%	248	96.88%	189	73.83%	143	55.86%
	Positive ROIs	50	100%	50	100%	48	96%	46	92%
	Negative ROIs	206	100%	198	96.12%	141	68.45%	97	47.09%

DDSM	Total patches	2300	100%	1397	60.74%	1074	46.70%	671	29.17%
	Positive patches	198	100%	185	93.43%	180	90.91%	161	81.31%
	Negative patches	2102	100%	1212	57.66%	894	42.53%	510	24.26%
	Total ROIs	766	100%	690	90.08%	528	68.93%	418	54.57%
	Positive ROIs	144	100%	142	98.61%	137	95.14%	131	90.97%
	Negative ROIs	622	100%	548	88.10%	391	62.86%	287	46.14%

**Table 4:** Influence of the breast density on the proposed detection algorithm.

*MIAS*					
Density	*F*	*G*	*D*	—	All
Proportion	44%	36%	20%	—	100%
Sens	90.9%	94.44%	90%	—	92%
FPI	1.77	2.05	2.1	—	1.94

*DDSM*					
Density	1	2	3	4	All

Proportion	7.69%	23.84%	36.92%	31.53%	100%
Sens	90%	96.77%	93.75%	92.68%	93.84%
FPI	1.2	2.0	2.08	2.75	2.21

The first row shows the quantity proportion of each subset with different densities on both MIAS and DDSM data sets. The metrics are Sens and FPI.

**Table 5:** Comparing the performance (Sens and FPI) of the proposed method with existing algorithms on the MIAS and DDSM data sets.

Algorithm	Data set	Sens	FPI
Wavelet processing and adaptive threshold [51]	MIAS	90.9%	2.35
Havrda and Charvat entropy and OSTU [52]	MIAS	93.2%	7.6
Adaptive median filtering and texture analysis [58]	MIAS	92.3%	2.75
Our method (Gestalt psychology)	MIAS	94%	2.16
Wavelet processing and adaptive threshold [51]	DDSM	91%	2.1
Kernelized supervised hashing [59]	DDSM	94%	4.1
Our method (Gestalt psychology)	DDSM	94.6%	2.66
